# Supplementary material for: Local Ablative Therapy Associated with Immunotherapy in Locally Advanced Pancreatic Cancer: A Solution to Overcome the Double Trouble?—A Comprehensive Review
Source: J Clin Med. 2022 Mar 31;11(7):1948. doi: 10.3390/jcm11071948 (PMC8999652; doi:10.3390/jcm11071948)
Supplement: Supplementary file 1 [file jcm-11-01948-s001.zip › jcm-1557497-supplementary.pdf]

Section S1

Search strategy PubMed

(Locally advanced[tiab] OR advanced[tiab] OR unresectable[tiab])

AND

(Pancreatic Neoplasms[Mesh] OR (Pancreas[tiab] AND cancer[tiab]) OR (Pancreatic[tiab] AND cancer[tiab]) OR (Pancreatic[tiab] AND adenocarcinoma[tiab]) OR (Pancreas[tiab] AND adenocarcinoma[tiab]))

AND

(Ablation Techniques[Mesh] OR Ablation Technique*[Tiab] OR Irreversible electroporation[Tiab] OR IRE[Tiab] OR electroporat*[tiab] OR radiofrequency ablation[tiab] OR RFA[tiab]OR microwave*[tiab] OR MWA[tiab] OR cryoablation[tiab] OR Laser*[tiab] OR Radiation[tiab])

AND

(Immuno*[tiab] OR Immune checkpoint *[tiab] OR Targeted therapy*[tiab])
